# Dynamics of fungal community during silage fermentation of elephant grass (*Pennisetum purpureum*) produced in northern Vietnam

**DOI:** 10.5713/ajas.18.0708

**Published:** 2019-07-01

**Authors:** Viet Ha Vu, Xiyang Li, Mengyuan Wang, Rongmei Liu, Guojian Zhang, Wei Liu, Baixue Xia, Qun Sun

**Affiliations:** 1 Key Laboratory of Bio-resource and Bio-control of the Ministry of Education, College of Life Sciences, Sichuan University, Chengdu, Sichuan 610064, China; 2 Department of Animal Science and Technology, North East College of Agriculture and Forestry, Quang Ninh 207620, Vietnam

**Keywords:** Elephant Grass, Fermentation, Fungal Community, High-throughput Sequencing, Silage

## Abstract

**Objective:**

This study aimed to gain deeper insights into the dynamic changes in spoilage fungi populations during fermentation and the influence of traditional additives on silage quality.

**Methods:**

Elephant grass (*Pennisetum purpureum*) was prepared without any additive (control), and with the addition of 0.5% salt, and 0.5% salt−0.2% sugar mixture. The fungal community was then determined using a classic culturing method and high-throughput sequencing at 0, 5, 15, and 60 days after ensiling.

**Results:**

The results showed that the fungal community of elephant grass silage varied significantly between the natural fermentation without any additive and the two additive groups. The diversity and relative abundance of spoilage molds in the control group were much higher than those in the two treatment groups (p<0.05). Three species of yeasts (*Candida* sp., *Pichia* sp., *Trichosporon* sp.) and four spoilage molds (*Fusarium* sp., *Aspergillus* sp., *Muco* sp. and *Penicillin* sp.) were the predominant fungi in elephant grass during natural fermentation from 0 to 60 days, which were found to be significantly decreased in salt and sugar additive groups (p<0.05). Meanwhile, the diversity and relative abundance of undesirable molds in the 0.5%-salt additive group were the lowest among all groups.

**Conclusion:**

Adding salt and sugar, particularly 0.5% salt, is a promising effective approach to reduce the amount of undesirable fungi thus, improving the silage quality of elephant grass in northern Vietnam.

## INTRODUCTION

Elephant grass, an excellent forage in the tropical and subtropical regions, is the major livestock feed in Vietnam, particularly for cattle, dairy and sheep [1]. When pasture is in poor quality or unavailable, the superiority of elephant grass silage is prominent due to its high nutrient quality, yield and adaptability [2]. The main purpose of ensiling is to keep forage available throughout the year. As elephant grass can be used as the main source of feed for ruminants because of its high nutritional value, it is significant in the development of sustainable agriculture.

Ensiling is a forage preservation process that occurs under anaerobic fermentation con ditions, which reduces the pH of the fermented product through a homofermentation process of major water soluble carbohydrates to lactic acid by lactic acid bacteria (LAB), thereby inhibiting harmful microorganisms and preserving nutrient content [3]. The nutritional availability of good quality silage is close to that of the raw materials and is also highly palatable. Feed intake, organic digestibility and effective energy of silage are also similar to the raw materials for ruminants. Fungal spoilage is one of the greatest risks to silage because fungal growth leads to loss of nutrients, dry matter and mycotoxin production, reduction in palatability and reduction in silage consumption, which leads to losses in animal performance [4]. Silage fermentation quality affects feed intake, milk composition and milk flavour. Management is the most important factor affecting silage fermentation [5]. However, poor storage conditions such as leakage of rainwater, excessive heat, moisture and lack of additional additives in silage can lead to undesirable mold contamination.

Generally, the ensiling process of elephant grass is defined by the following steps: initial stage (0 to 5 days): the elephant grass is harvested, chopped, loaded into a silo before compacted and sealed to exclude air; middle fermentation (5 to 15 days): grass consumes the oxygen and there is a break down of carbohydrates; end fermentation (15 to 60 days): the silage is fermented in anaerobic conditions for several months till the silos are reopened for animal feeding.

Silage is vulnerable to contamination by spoilage molds, as ensiled materials are excellent substrates for the growth of fungi [6]. Moreover, as a product of anaerobic fermentation of cut plant material, silage contains high levels of microorganisms that can be regarded as beneficial or undesirable [7]. Undesirable fungi, such as yeasts and molds, are responsible for silage degradation. However, the species and levels of these fungi vary depending upon the conditions of ensiling in any particular batch of silage [7,8]. Epiphytic yeasts presented in silage can convert WSC into carbon dioxide (CO_2_) and alcohol, which can provide silage fermentation with the perfect conditions. Meanwhile, accumulating excess alcohol is known to be toxic and impairs silage quality, leading to a potential decrease in feed intake. On the other hand, ensiling can act as a vector for several undesirable microorganisms which can impair silage preservation and affect animal performance, or even animal and human health [6]. Yeasts and molds were able to produce many secondary metabolites, including mycotoxins, which remained in the silage even after the fungi has disappeared [9]. Chronic exposure to low levels of mycotoxins through contaminated animal feeds would typically lead to non-specific symptoms such as immune system impairment, increased infections, and metabolic and hormonal imbalances [10]. The transfer of toxins to dairy and meat products is a potential risk to humans. However, data on the microbial communities involved in tropical grass ensiling are unavailable or largely concerned with questions regarding bacteria and elephant grass quality changes during the silaging process. Therefore, due to the impact of fungi on silage quality, identifying the major spoilage molds and yeasts in elephant grass silage is pressing.

In order to avoid silage degradation from possible patho gens, additives have often been used to improve the silage process and quality. Additives can have significant effects on the quality of silage by enabling the production of more organic acids and greater reductions in pH, as well as effectively inhibiting the activity of molds [11]. In northern Vietnam, with many years of production experience, local farmers have used common additives, including salt and sugar, to improve the quality and taste of elephant grass silage. Salt (NaCl) is one of the most important additives for ensiling and preserving food as it can not only inhibit the growth of harmful bacteria, but also improve the fermentation quality of silage [12]. Now many reports have proposed that adding different proportions of sugar can effectively improve silage quality [13]. In this study, samples were taken from farms in northern Vietnam, where local farmers find that the addition of salt and sugar can effectively maintain nutrient levels and extend storage time. Small-scale farms usually use 0.5% salt in grass silage based on their experience. Some farmers also added a lower concentration 0.2% of sugar with 0.5% salt in silage. It is not currently known which additives are most beneficial for inhibiting undesirable yeasts, molds and pathogens. Until now, few studies have addressed this problem.

Therefore, the aims of the present work were to investi gate the dynamic changes in fungi and identify the major spoilage molds of elephant grass silage in the northern region of Vietnam during the fermentation process after treatment with salt and salt-sugar additives.

## MATERIALS AND METHODS

### Sample collection

The samples were collected in Ha Noi, Vietnam where silage practice was originally developed. A total of 30 samples of elephant grass silage were collected from 27 different silos with 3 kinds of additives, during 3 periods. Samples were treated with nothing (natural fermentation), 0.5% salt, and 0.5% salt combined with 0.2% sugar at day 5, day 15 and day 60. Three extra samples of fresh elephant grass were used as samples on day 0. Samples were obtained by collecting material from four different sections of each silo; the upper layer, the lower layer, the laterals and the central part. A 1 kg sample of silage material was taken from each section. All data represents a mixture of these four different positions as a 200 g sample from each sampling site was obtained. Each combined silage sample was plated out three times. Samples were collected in sterile packages and stored at −20°C prior to DNA extraction.

### Yeast and mold counts and isolation

Total fungal counts of samples were done according to the method described by Pitt and Hocking [14]. Basically, 10 g of each sample was aseptically blended in 90 mL (0.1%) peptone water. A 1 mL aliquot was transferred to a test tube containing 9 mL of peptone water to make a 10^−1^ dilution. Serial dilutions until 10^−5^ were made and 0.1 mL aliquots were inoculated, in duplicate, on the surfaces of Potato Dextrose agar (1% Chloramphenicol) and Di-Chlorine Rose Bengal Chloramphenicol agar. All experiments in each treatment group were prepared in three technical replicates. The plates were then incubated at 28°C for 6 to 8 days. Only plates containing 15 to 150 colony-forming units (CFU) were used for counting and the results were expressed as CFU per gram of sample (CFU/g). Following incubation, all the isolates were sub-cultured onto Czapek yeast agar (CYA), malt extract agar (MEA) and glucose-peptone-yeast extract agar (GPY), pH 3.7 to 4.5. Both CYA and MEA were used for filamentous fungi while GPY was used for yeasts. Molds and yeasts were identified by 18S rRNA sequencing. The universal primers used were ITS1 5′-TCCGTAGGTGAACCTGCGG-3′ and ITS4 5′-TCCTCCGC TTATTGATATG-3′ and the results were expressed as isolation frequency of the fungal genera.

### Microbial diversity analysis by high throughput sequencing

#### Microbial DNA extract

Fungal DNA was extracted with the Biospin Plant Genomic DNA Extraction Kit (Bioer Technology Co., Ltd, Hangzhou, Zhejiang, China) using a traditional culture method. For the molecular analysis of microbial communities, genomic DNA was extracted using the Power Soil DNA Isolation kit (Mobio, Diego, CA, USA). The aliquoted DNA samples were kept at −20°C until further analysis. Microbial DNA extraction was performed according to the manufacturer’s instructions.

#### Polymerase chain reaction amplification and sequencing

To amplify the V3-V4 region of the 18S rRNA gene for Illumina sequencing, universal primers ITS1F: 5′ - CTTGGTCA TTTAGAGGAAGTAA - 3′ and ITS2R: 5′ - GCTGCGTTC TTCATCGATGC - 3′ were used. The polymerase chain reaction (PCR) was performed in a total reaction volume of 20 μL: H_2_O 13.25 μL, 10×PCR, Ex Taq Buffer 2.0 μL, DNA template (100 ng/mL) 0.5 μL, prime 1 (10 mmol/L) 1.0 μL, prime 2 (10 mmol/L) 1.0 μL, dNTP 2.0 μL, and Ex Taq (5 U/mL) 0.25 μL. After an initial denaturation at 95°C for 5 min, an amplification was performed with 30 cycles of incubation for 30 s at 95°C, 20 s at 58°C, and 6 s at 72°C, followed by a final extension at 72°C for 7 min. The amplified products were purified and recovered using the 1.0% agarose gel electrophoresis method. Finally, the library construction and sequencing steps were performed by Beijing Biomarker Technologies Co. Ltd. (Beijing, China)

### Statistical analyses

We identified possible chimeras by employing UCHIME, a tool included in the mothur package (http://drive5.com/uchime). The denoised sequences were clustered using the QIIME UCLUST module and tags with similarity ≥97% were regarded as an operational taxonomic unit (OTU). Each OTU was filtered with a threshold of 0.005% of all sequences. The alpha diversities of samples, mainly of the Shannon, Simpson index, Chao1 richness estimator and the Good’s coverage, were determined using MOTHUR software (ver. 1.30.1, http://www.mothur.org/wiki/Classify.seqs). To identify OTUs whose abundance differed significantly between the three sampling sites, an analysis of variance was conducted, in which the p-value was adjusted using the Bonferroni correction. Taxonomy was assigned to all OTUs by searching against the Silva databases (Release119, http://www.arb-silva.de) using the principal component analysis (PCA) classifier within QIIME.

Data are expressed as mean±standard deviation for each assay and analyzed using paired sample t-test as part of the SPSS 19.0 software package. Statistical significance was declared at p<0.05. Data were compared using analysis of variance according to the general linear model procedure of SPSS. Pearson correlations were used for testing the correlation between variables. Figures were drawn using OriginPro 8 and Photoshop CS5.

## RESULTS AND DISCUSSION

### Fungal diversity characteristics

**Figure 1:**
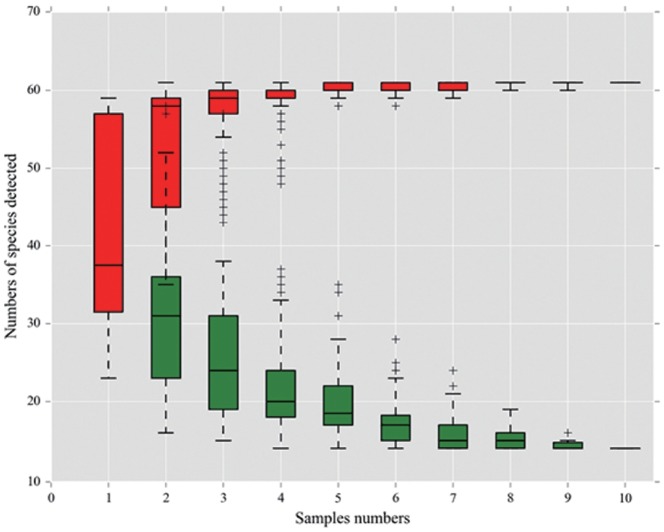
Species accumulation curve performed using the Biomarker biocloud platform (www.biocloud.org). The red rectangle is the accumulation curve and the green rectangle is the common amount. As the sample number increased, the curve reached a plateau and there was no further obvious increase in the number of species, which indicated that the sample volume in our study was large enough to reflect the species richness.

Table 1 describes the total yeast and mold counts (CFU/g) in elephant grass silage samples using the traditional method. The number of fungi was high in the control group and low in the two additive groups. Yeast and mold counts were significantly different between the control and two additive groups (p<0.05). The number of fungi was high in the control and low in the additive group. Possible reasons were that the growth of fungi was inhibited by salt, which enhanced fermentation. Sugar provided carbohydrates for LAB that increased accumulation of lactic acid, resulting in a low pH that can inhibit the growth of molds and yeasts [15]. Decreased pH and increased lactic acid resulted in a lower number of fungi in the sugar group. Many reports have shown that counts of molds and yeasts were also lower in salt-treated or sugar-treated silage, which tended to have a higher dry matter content and lower pH compared with control silage [16]. Our data also showed that fungal community richness decreased when salt or salt plus sugar were added compared to the control. Theoretically, mold counts in the salt-sugar additive group should have been lower than the salt additive group. However, in this study, mold counts in the salt additive group were significantly (p<0.05) lower than salt-sugar additive group. The reason may be that the amount of sugar added (0.2% sugar on fresh material basis) may be not enough to promote epiphytic LAB activity. On the other hand, molds may utilize sugar to grow and compete with other microorganisms during aerobic fermentation. Meanwhile, to gain deeper insights into fungal community dynamics in the ensiling of elephant grass, high-throughput sequencing was used in this study. Based on the sample number and the OTUs, a species accumulation curve for all samples was calculated (Figure 1). In this study, the curve reached a plateau and there was not an obvious increase in the number of species with an increasing number of samples, indicating that the sample number in our study was large enough to reflect species richness. The sampling completeness was evaluated by the Good’s coverage values which were approximately 0.99 in all samples indicating that most of the fungal community in the samples were adequately captured.

In order to identify the dynamic changes of fungi with the lengthening of fermentation time by different additives, fungal community diversity was assessed by sequencing the fungal 18S rRNA V3+V4 regions at different fermentation times with two additives (Table 2). After removing the low-quality sequences, 2,399,340 reads with an average length of 200 bp per sequence were subjected to the following analysis. Furthermore, these reads were clustered into 216 core OTUs based on 99% similarity level (equal to the species level). The Chao1 index indicates the richness of the microbial community. Chao1 also indicates the number of species and therefore the richness of the fungal community. The larger the Chao1, the greater the number of OTUs and the greater the richness of the community contained in the group. Shannon indices, which indicate the diversity of the fungal community were negatively affected by the additives [17]. The Chao1 index showed a sharp increase (from 157-184-200-210 at 0, 5, 15, and 60 days, respectively) in the richness of the fungal community during the elephant grass silage under natural conditions (control group). However, after adding salt or salt-sugar, the Chao1 estimator varied 157-192-88-193 and 157-177-119-136, respectively. Based on these data, we found that, after an initial increase, fungal community richness drastically decreased in the salt and salt-sugar additive groups. This result was also proved by traditional methods (Table 1). Moreover, the salt additive group had much lower fungal community richness than the salt-sugar additive group and control. Conversely, at the end of the fermentation period the salt-sugar additive group exhibited lower fungal community richness than the salt additive group.

**Figure 2:**
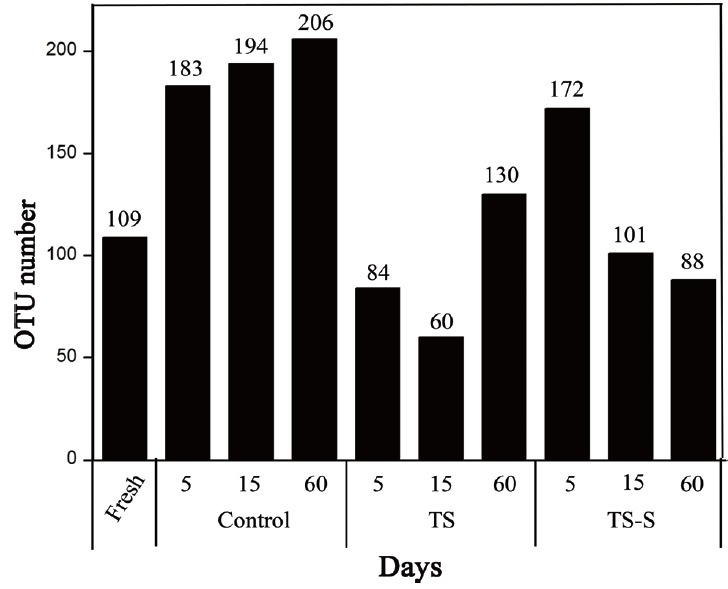
Changes in the number of fungal OTUs during elephant grass ensiling with different additives and on different days. Higher diversity of molds and yeasts in the control were found compared to the two additive groups. OTUs, number of operational taxonomic unit. Control, natural fermentation; TS, treated with 0.5% salt; TS-S, treated with 0.5% salt and 0.2% sugar.

In addition, changes in the number of fungus OTUs during elephant grass ensiling with different additives and on different days (Figure 2) were investigated. Fungal diversity was significant different only at genus and species levels between the additive group and the control (p<0.05), but no significant differences could be found between the two additives groups at all levels. This illustrates that salt and sugar can inhibit the growth of fungi in comparison to the control, but the differences between the two additive groups were not significant. Consistent with the number of Chao1, the total number of OTUs in the salt additive group was much less than the control and salt-sugar additive group in the middle of the fermentation process. Therefore, during this period, the number and species of fungi changed a great deal. In conclusion, the combined traditional culture method and high-throughput sequencing confirmed that adding a salt or salt-sugar mixture can effectively decrease fungal community diversity.

### Principal component analysis

**Figure 3:**
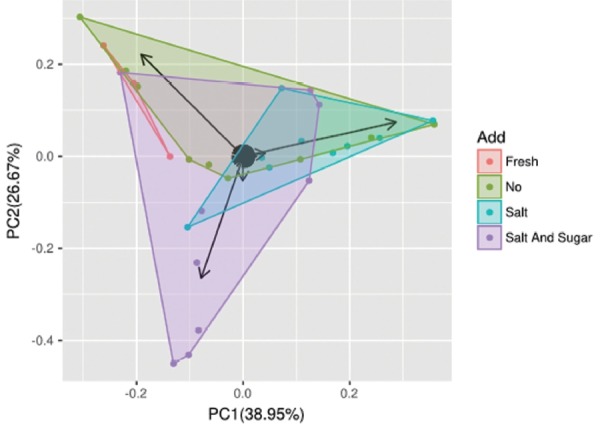
Principal component analysis of sample silage with different additives. The number of overlapping OTUs with different additives was very low. OUT, number of operational taxonomic units. Component 1 and component 2 account for 38.95% and 26.67% of the total variance, respectively and 65.62% of the contribution.

The dynamic variance of fungal diversity with different additives can be demonstrated by PCA. As shown in (Figure 3), component 1 and component 2 account for 38.95% and 26.67% of the total variance, respectively, and 65.62% of the contribution. The number of overlapping OTUs with different additives was very low. Fresh fermentation can be easily separated from other treatment silage. It was revealed that the fungal communities differed significantly between the fresh and additives silage samples (no, salt, salt-sugar). Many fungi contributed to influence elephant grass silage. The arrows in the (Figure 3) show the main contribution of fungi to each principal component. In this figure, all arrows represent *Candida*, which was the principal fungal factor affecting the fungal community structure with different additive treatments. This is verified below.

### Heat map

**Figure 4:**
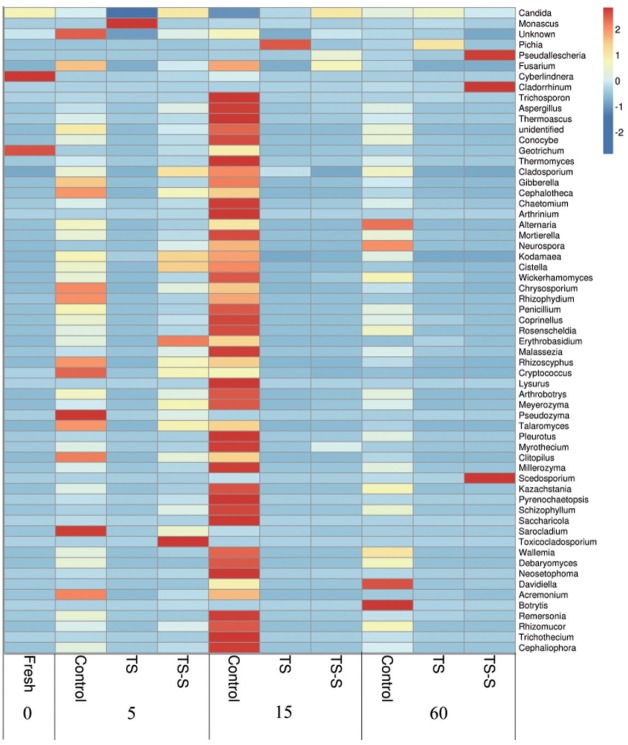
Heat map of fungal relative abundance at the genus level. Silage samples are labeled with Latin letters indicating the type of treatments (control, natural fermentation; TS, treated with 0.5% salt; TS-S, treated with 0.5% salt and 0.2% sugar) and Arabic numerals mean the day of ensiling. The deepest red color signifies high relative abundance, indicating that fungi were very active during this stage. The dynamics of molds and yeasts in the control were found to be different to the two additive groups.

The relative abundance of fungi with the lengthening of storage time and the influence of different additives was compared by heat map analysis at genus level (Figure 4). The deepest red color signifies high relative abundance, indicating that fungi were very active in this stage. Based on the data, the dominant fungi were molds and yeasts in all samples. Among them, four dominant phyla of molds were identified in elephant grass silage, including *Chytridiomycota*, *Ascomycota*, *Zygomycota*, and *Basidiomycota*. Small differences were shown among samples at phylum level in all groups. However, at the species level the dynamic of molds and yeasts in control were found to be different from the two additives groups. Also, a much higher diversity of fungi was found in the control (Figure 4).

*Cyberlindnera* and *Geotrichum*, a yeast and mold respectively, had high relative abundance in fresh elephant grass. *Geotrichum* is the dominant mold in maize silage [18]. On day 5, the predominant fungi changed to *Pseudozyma*, *Sarocladium*, *Cryptococcus*, and *Rhizoscyphus* in the control group due to initial high oxygen content and high WSC in elephant grass silage. Most of the fungi in the data were molds commonly detected in other plant silage such as maize silage [19], and so on. Their fast growth indicates that the elephant grass had begun to rot. However, after adding salt, the relative abundance of *Pseudozyma*, *Sarocladium*, *Cryptococcus*, and *Rhizoscyphus* were very low on day 5, and few species of spoilage molds existed. The growth of spoilage molds and yeasts during the early stage would significantly affect the quality of elephant grass silage. After the addition of salt-sugar, at the beginning of the fermentation process, the growth of molds and yeasts were significantly inhibited by the salt. Therefore, the relative abundance of putrefactive organisms was more than that of the salt additive group, such as *Toxicocladosporium* and *Erythrobasidium*, but far less than the control group. As silage progressed, oxygen content and pH decreased. Fungi can continue to grow in these conditions. During the middle stage, on day 15, decay was worse than before in the control group, with a high relative abundance of putrefactive organisms found, including *Pseudozyma*, *Fusarium*, and *Penicillium*. This is in contrast with the additive groups. Few red spots were found in the additive group, explaining why putrefactive organisms were not active after being treated with salt or salt-sugar. The dominant genus in the salt additive group was *Pichia* due to its salt resistance [12] and acid tolerance which can survive in salty environments. However, the overall level of spoilage molds was very low in the salt additive group, which may indicate good silage quality. Particularly at the end of fermentation, the advantage of adding salt was clear. No red spots were found in the salt-sugar additive group indicating that addition of the salt-sugar additive could inhibit the growth of putrefactive organisms effectively in the middle silage of elephant grass at the end of the fermentation period (60 days). In the salt-sugar additive group, the number of species and the relative abundance of molds and yeasts, such as *Pseudallescheria*, *Cladorrhinum*, and *Scedosporium*, began to increase. *Cladorrhinum* and *Scedosporium* are infrequently associated with human and animal opportunistic infections. This may be because sugar offers the nutrients for fungus growth. However, the most common putrefactive organisms remained active in the control group, including *Botrytis* and *Alternaria*.

In view of the results, the diversity of the fungal community was remarkably increased during the silaging process in the control group. Moreover, the diversity and relative abundance of putrefactive organisms also maintained high levels, more so than the additive groups. This indicates that the high diversity and relative abundance of putrefactive organisms resulted in the low quality of elephant grass silage. Salt and salt-sugar treatments can significantly inhibit the growth of fungi. In particular, the addition of salt could improve the quality of elephant grass silage significantly.

### Community dynamics of the predominant undesirable microorganisms

Elephant grass has been viewed as a difficult crop to ensilage, primarily because of its high water content, high water-soluble carbohydrates and low oxygen content. These conditions are conducive to the development of undesirable microorganisms, including yeasts and molds which are responsible for silage degradation with potential negative effects on animal and human health. The predominant undesirable microorganisms, including yeasts and molds, were similar across the two methods; however, more fungi were found using a high throughout method. Of the undesirable microorganisms found in silage, yeasts are the most important group as they are involved in aerobic spoilage, during the aerobic phase at the beginning of ensiling. Silage oxygenation restarts yeast organic acid metabolism pathways (succinic, citric, and lactic acids) inducing a pH increase and allowing for the growth of less acid-tolerant microorganisms. On the other hand, epiphytic yeasts present in silage can convert WSC into CO_2_ and alcohols, which are known to be toxic to the liver. Alcohols can increase dry matter loss, decrease nutritional value, rapidly corrupt silage and decrease feed intake [20].

**Figure 5:**
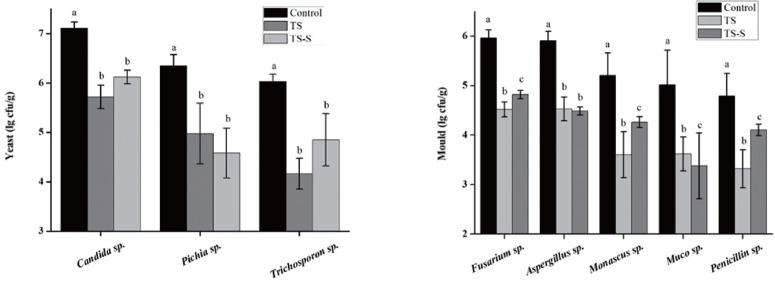
Predominant yeast (A) and mold (B) counts (CFU/g) found in elephant grass silage samples by traditional culture method. Three genera of yeasts (*Candida* sp., *Pichia* sp., *Trichosporon* sp) and five genera of molds (*Fusarium* sp., *Aspergillus* sp., *Monascus* sp., *Muco* sp. and *Penicillium* sp.) were most frequently found. CFU/g, colony-forming units per gram of sample. Control, natural fermentation; TS, treated with 0.5% salt; TS-S, treated with 0.5% salt and 0.2% sugar. Means within a column with different lowercase letters (a–c) differ from each other at p<0.05.

**Figure 6:**
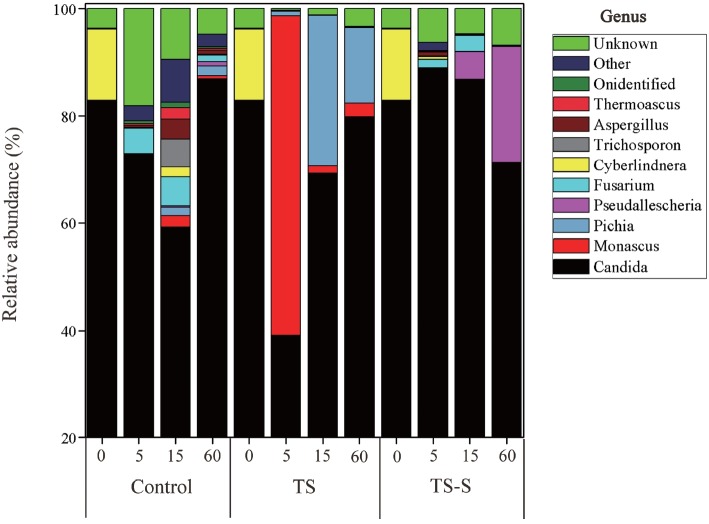
Relative abundance of the 10 most commonly isolated molds at the genus level. Silage samples are labeled with Latin letters indicating the type of treatment (control, natural fermentation; TS, treated with 0.5% salt; TS-S, treated with 0.5% salt and 0.2% sugar) and Arabic numerals mean the day of ensiling.

In order to improve elephant grass silage preservation and guarantee the quality of this animal feed, the species of the predominant putrefactive organisms during elephant grass fermentation were identified using traditional culture methods (Figure 5) and high throughout methods (Figure 6). Yeasts and molds are considered to be the most important putrefactive organisms found in silage [21]. As shown in (Figure 5), three kinds of yeasts and five kinds of molds were most frequently found. As expected, *Candida* sp., *Pichia* sp., and *Trichosporon* sp. were the top-ranked yeasts in both treatments. Furthermore, the number of each in all treatment groups was significantly different to the control group. The predominant molds identified were *Fusarium* sp., *Aspergillus* sp., *Monascus* sp., *Muco* sp., and *Penicillium* sp. Among them, *Fusarium* sp., *Aspergillus* sp., *Muco* sp., and *Penicillium* sp. were the most common undesirable molds [22]. The number of these molds was significantly different to that seen in the control group. Differences were also present between the inter- and intra-group in the treatment group, except for *Aspergillus* sp., indicating that adding salt and sugar can inhibit the growth of yeasts and molds. The diversity and relative abundance of mold after salt and sugar addition were also significantly different (p<0.05). Thus, the addition of salt and sugar could be a good approach to improve the quality of elephant grass silage.

Using the high throughout method, ten commonly oc curring OTUs at the genus level, isolated from the fungal community, were selected to identify the main spoilage fungi. The top ten fungi based on their relative abundance at the genus level are listed in (Figure 6). This shows that the predominant fungi in elephant grass silage shifted significantly between the spontaneous fermentation and two additive groups during the fermentation period. *Candida* was the predominant yeast in all groups and had a high relative abundance (over 65%). On day 0, the relative abundance of *Candida* reached 83.44%. One exception was *Monascus*, whose relative abundance (59.61%) far exceeded *Candida* (39.13%), during the initial stage of the salt additive group. It may be that after adding salt, fermentation conditions changed so that the growth of *Candida* was inhibited and *Monascus* was able to quickly grow due to its resistance to heat, alcohol, lactic acid and high salt levels [20]. Furthermore, *Monascus* can survive under reduced oxygen levels when NaCl concentration levels range from 2% to 10% in the Greek table olive industries [23]. The relative abundance of *Monascus* descended rapidly when fermentation conditions became incompletely anaerobic. Overall, in the salt additive group, the relative abundance of *Candida* (39.13%) was far less than the control, even lower than the salt-sugar additive group (83.44%). This can decrease the production of alcohols to reduce the risk of liver toxicity and improve silage quality. The fungi found by traditional cultivation and high-throughput analysis are slightly different, probably because some fungi cannot grow or grow slowly on culture medium thus less species of fungi were shown in traditional approach. Yeasts accounted for a large proportion in traditional method, so did in high-throughput analysis. Therefore, fewer species of molds presented among the dominant fungi species in high-throughput results.

In the early stages of fermentation, some oxygen remained for microorganism reproduction, while the respiration of plants made the type of microorganisms change dramatically. As the oxygen content declined sharply and the temperature decreased after the oxygen was used up, the available nutrients for the microorganisms gradually diminished. LAB can convert carbohydrates into carbon dioxide, ethanol and organic acids that lower the pH, which together with the anaerobic conditions, prevent the growth of spoilage organisms, such as fungi and other bacteria [24]. However, in natural fermentation, the number of epiphytic LAB was initially very low. Along with fermentation, LAB composted insufficiently (heterofermentation) which was not enough to reduce pH value and suppress undesirable microorganisms [25]. On day 0, the most abundant fungi were associated with the *Cyberlindnera* (13.28%) and the *Fusarium* (0.17%). Moreover, *Aspergillus* accounted for as little as 0.01% in the beginning. On day 5, *Fusarium* sp. was the most frequent spoilage mold in elephant grass silage, followed by *Aspergillus* sp., *Penicillium* sp., *Trichosporon* sp., and *Cladosporium* [22], all of which are mycotoxigenic fungi [26]. In the control group on day 15, the most abundant spoilage molds were *Fusarium* sp. (5.47%), *Trichosporon* sp. (5.22%), and *Aspergillus* sp. (3.68%). Under these conditions, spoilage molds reproduce quickly and gradually deteriorate the elephant grass. Therefore, natural fermentation silage resulted in unsatisfactory preservation due to the relatively low content of lactic, acetic and butyric acids, the high pH, and the promotion in the breakdown of protein to NH_3_-N [27]. Based on this, it would be necessary to add LAB with the salt and sugar to further improve the silage quality of elephant grass.

Additives can have significant effects on the quality of si lage by enabling the production of more organic acids and greater reductions in pH, as well as effectively inhibiting undesirable microorganisms [11,28]. Compared to the control group, the fungal community changed after the addition of salt or salt sugar. In the salt-sugar group, *Pseudallescher* was dominant in the middle and later stages, which was linked to a decrease in the protein content and nutritional value of the silage for the production of biogenic amines, including putrescine, cadaverine and tyramine in silage [29]. *Fusarium* and *Aspergillus* always showed the lowest relative abundance and could not be detected at the end of the fermentation period. Few spoilage molds were found in the salt additive group. Overall, using additives modified the structure of fungi and reduced the diversity and relative abundance of undesirable microorganisms compared to the control group.

In theory, the relative abundance of molds and yeasts in the salt-sugar additive group should decrease, in contrast to the control and salt additive groups. While salt acts to sterilize, sugar can increase the water soluble carbohydrate content which is converted into organic acids, mainly lactic acid by LAB, thereby reducing pH. It has been suggested that salt and sugar addition could effectively inhibit the growth of harmful spoilage fungi and improve the silage quality of elephant grass. However, in this study, undesirable fungi in the salt additive group were much less than that found in the salt-sugar additive group. There are two primary reasons for this result: i) poor sealing in the salt-sugar additive group prolonged oxygen consumption; and ii) molds and LAB in the salt-sugar additive group grew competitively for sugar. One study showed that with the addition of 3% molasses, dwarf elephant grass had a higher content of lactic acid, dry matter and crude protein and a lower pH compared with the control perhaps 0.2% sugar added to elephant grass silage may be not enough.

In conclusion, the addition of 0.5% salt and 0.5% salt−0.2% sugar can effectively improve the silage quality of elephant grass. However, the total fungal counts in this study were above 1×10^4^ CFU/g, which exceeded the quality standard for animal feeds [30] (Table 1). In Table 1, the total counts of yeasts and molds present in the salt and salt-sugar additive groups were 7.9×10^5^, 8.7×10^4^, 1.9×10^6^, and 1.7×10^5^, respectively. Hence, more research is needed to further decrease the number of fungi in silage. Recently, many papers reported that the addition of LAB could effectively improve the silage quality of barley silage [17] and maize [21] and reduce the abundance of undesirable microorganisms. It is necessary to use LAB and salt as additives to further improve the silage quality of elephant grass in the next stage of investigation.

## CONCLUSION

We found that common additives used for traditional silage, including salt and sugar, could inhibit the growth of undesirable yeasts, molds and pathogens. Based on our experimental data, the growth of harmful fungi was significantly inhibited in the 0.5% salt group compared to the 0.5% salt−0.2% sugar group. Therefore, adding 0.5% salt is more effective than adding 0.5% salt−0.2% sugar. Next, we plan to use a combination of LAB, sugar and salt to investigate whether these additives can further improve the silage quality of elephant grass.

## Figures and Tables

**Table 1:** Total yeast and mold counts (CFU/g) found in elephant grass silage samples collected from Ha Noi, Vietnam

Treatment	Yeasts (CFU/g)±SD	Molds (CFU/g)±SD
Control	1.7×10^7^±4.5×10^6^ b	2.4×10^6^±1.3×10^6^ b
TS	7.9×10^5^±7×10^5^ a	8.7×10^4^±1.4×10^4^ a
TS-S	1.9×10^6^±7.4×10^5^ a	1.7×10^5^±3.9×10^4^ c

Silage samples were labeled with Latin letters indicating the type of treatment (Control, natural fermentation; TS, treated with 0.5% salt; TS-S, treated with 0.5% salt and 0.2% sugar); CFU/g, colony-forming units per gram of sample; experimental results were subjected to one-way analysis of variance followed by independent t-test at p<0.05 using SPSS 19.0 software.

a,b,c Means within a column with different superscripts differ (p<0.05); ±SD, standard deviation. The number of fungi was high in the control group and low in the two additive groups. Yeast and mold counts were significantly different between the control and two additive groups (p<0.05). Mold counts in the salt additive group were significantly (p<0.05) lower compared with the salt-sugar additive group.

**Table 2:** Fungal community diversity of elephant grass silage with different additives at different storage time

Treatments	Days	Reads	Length	OTUs	Shannon	Simpson	Chao 1	Coverage
Control	0	239,664	200	109	1.38	0.39	157	0.99
	5	239,531	211	183	1.66	0.48	184	1.0
	15	239,892	217	194	3.21	0.08	196	1.0
	60	239,730	191	206	1.30	0.63	210	1.0
TS	0	239,664	200	109	1.38	0.39	157	0.99
	5	240,303	218	84	1.09	0.43	192	0.99
	15	240,577	176	60	0.98	0.50	88	0.99
	60	240,593	180	130	1.63	0.27	193	0.99
TS-S	0	239,664	200	109	1.38	0.39	157	0.99
	5	239,915	192	172	1.71	0.30	177	1.0
	15	239,009	182	101	1.81	0.20	119	0.99
	60	240,126	182	88	1.36	0.34	136	0.99

Silage samples were labeled with Latin letters indicating the type of treatment (control, natural fermentation; TS, treated with 0.5% salt; TS-S, treated with 0.5% salt and 0.2% sugar); length, average read length (base pair); OTUs, number of operational taxonomic units.

The alpha diversities of samples, mainly of the Shannon, Simpson index, Chao1 richness estimator and the Good’s coverage, were created using MOTHUR software (ver. 1.30.1). To identify OTUs whose abundance differed significantly between the three samples, an analysis of variance was conducted.
